# Bibliography

**Published:** 1890-09

**Authors:** 


### BIBLIOGRAPHY.

IRREGULARITIES OF THE TEETH AND THEIR TREATMENT. By EUGENE S. TALBOT, M.D., D.D.S. Second Edition. 234 Illustrations. P. Blakiston, Son & Co., Philadelphia, 1890.

It is somewhat remarkable that the "Treatment of Irregularities" should have called forth in recent years a series of works from several of the best writers in the dental profession, while other, and seemingly more important, branches have been either wholly neglected or imperfectly described. This may be explained by the fact that it is one of the most difficult of subjects, and hence has an attraction for certain minds; and, further, the principles and practice governing it have received but little attention in the text-books of former years. The result of this extended labor has been very satisfactory, as this very difficult subject has at last been placed on a scientific basis, creditable alike to the authors of the several works and an honor to a young profession.

The author of the present work has devoted years to the study of the principles governing the changes in development of teeth, and must be regarded as an authority on all points connected therewith.

In his introduction he says, "In presenting his views regarding the etiology of irregularities of the teeth and their correction, the author lays particular stress on underlying principles." That he is correct in this must be admitted by all with any experience. He has, therefore, devoted a large portion, indeed almost the entire book, to this, leaving, it may be thought by some, a too meagre portion to treatment.

The book contains 261 pages, divided under the head of "Etiology" into twelve chapters, and under "Treatment" into six. Chapters III. to VI. under the first head (Etiology) embrace the conclusions of the author after years of labor, and are scarcely to be overestimated. These should be read with care, as they abound with facts of the highest importance. Whether we agree or not with Dr. Talbot's conclusions, his facts deserve the closest attention. "Local Causes" cover twenty-six pages. The following quotation from page 145, relating to the extraction of the first permanent molar, deserves to be read and pondered by those who sacrifice these teeth with an indifference that borders on criminality. Coming from such an authority, it should have due weight with this class.

"The wholesale extraction of the first permanent molar in the past has, no doubt, caused arrest of development of the alveolar process as well as of the maxillary bones, for the process and jaws depend for their development largely on the functions of the teeth, their articulation and their motion stimulating nutrition and enlarging the arch.

"Some of the older dentists, whose skill is the result of routine rather than knowledge, are still to be found extracting four sound molars without the least thought of the consequences. . . . The author has observed in many cases the want of development of the alveolar process, and sometimes of the jaws, from the extraction of those teeth. This assertion is verified in those cases where the germ has not developed and the tooth is missing. More marked instances are those where three or four germs are wanting. . . . The horizontal portion of the lower jaw will be but imperfectly developed, because function, one of the most important means of development, is lost, and insufficient room is left for the second and third molars."

Space will not permit a more extended notice of this valuable book. It is a marked advance over the first edition, and will be invaluable as reference to the future investigator into the causes that underlie irregularity.

The practical man who cannot perceive a value in anything unless it details minutely manipulations in any particular branch, will rise from its perusal with a sense of disappointment; but this superficial view cannot be entertained by those who appreciate the fact that nothing of any practical value can be attained until there is a mastery of the principles governing the work. To the latter class Dr. Talbot's book will be a welcome addition to the repertory of records, happily now rapidly increasing.

A System of Oral Surgery. By James E. Garretson, A.M., M.D., D.D.S. Fifth Edition. J. B. Lippincott Company, 1890.

The fact that a fifth edition of this work of Professor Garretson has been required is substantial evidence of its value. It has long been a standard work of reference, notwithstanding that its size has militated against its use as a text-book. It has been, in the opinion of many, overloaded with matter properly belonging to works on practice. Notwithstanding these admitted defects, it is the best work on Oral Surgery we have or are likely to have for some time to come.

THE THERAPEUTICAL APPLICATIONS OF PEROXIDE OF HYDROGEN AND GLYCOZONE. By CHARLES MARCHAND, Chemist. New York, 1890.

This monograph, prepared, as it has been, to introduce the hydrogen-peroxide as manufactured by the author, is, nevertheless, a valuable contribution on this subject, giving in a condensed form matter of value to every dentist.

While this agent is extensively used, it is doubtful whether the large majority fully appreciate its value. As a cleaner of pus-pockets it has no superior, and it should be in universal use. The subject is thoroughly treated in this pamphlet, and we can heartily recommend the profession to procure it and make use of the agent.

